# Clinical characteristics of depression among adolescent females: a cross-sectional study

**DOI:** 10.1186/1753-2000-4-26

**Published:** 2010-10-10

**Authors:** Afaf H Khalil, Menan A Rabie, Mohamed F Abd-El-Aziz, Tarek A Abdou, Amany H El-Rasheed, Walaa M Sabry

**Affiliations:** 1 Institute of Psychiatry, Faculty of Medicine, Ain Shams University, Cairo, Egypt

## Abstract

**Background:**

Adolescents rarely seek psychiatric help; they even hesitate to disclose their feelings to their parents. However; the adolescents especially the females experience depressive symptoms more frequently than general population. Do they experience classic depressive symptoms? Are there symptoms specific to this subpopulation?

**Aim of the study:**

Through this study, the authors aimed to estimate the prevalence of depressive disorders in Egyptian adolescent female students. They also expected a characteristic profile of symptoms for the adolescent females. However available literature provides no guidance in the description of this profile of symptoms.

**Methods:**

A number of 602 adolescent females were interviewed, and subjected to General Health Questionnaire (GHQ); Children Depression Inventory (CDI), Structured Clinical Interview for DSM-IV Axis-I Disorders (SCID-I), then Hamilton Rating Scale for Depression (Ham-D). Results were analyzed by the use of SPSS-15.

**Results:**

The study revealed the prevalence of depression in the sample of the study to be 15.3% (measured by CDI), and 13.3% (measured by SCID-I). Fatigue was the most common presenting depressive symptom (81.3%), in addition to other emotional, cognitive and physiological symptoms. Suicidal ideations were the most common suicidal symptoms in depressed adolescent females (20%), with 2.5% serious suicidal attempts.

**Conclusions:**

The somatic symptoms were by far the most common presenting symptom for female adolescents suffering from depressive disorders. Depressive phenomena including unexplained fatigue, decreased energy, psychomotor changes, lack of concentration, weight changes and suicidal ideations may be the presenting complaints instead of the classic sad mood.

## Background

Depression is the most common mental disorder among adolescents with prevalence rates ranging from 15-20% among adolescents between the age of 14-19 years [1], and it is believed to be a major contributing factor in adolescent suicide [2]. Moreover, depressive disorders are significantly more common in females than in males, with lifetime prevalence of 14.1% for females and 8.6% for males [3]. Some epidemiological, community and clinical studies have shown that girls typically have been found to display higher levels of depressive symptoms than boys [4]. This has been attributed to genetics, increased prevalence of anxiety disorders in females, biological changes associated with puberty, cognitive predisposition and sociocultural factors [5].

Few Egyptian studies were conducted to investigate the prevalence and symptomatology of adolescent depression. In a study involving a sample of primary and preparatory schools in the city of Alexandria 10.3% of pupils demonstrated depressive scores, which were highest among the oldest age group (20.3%). Girls had higher depressive scores when they were compared with boys [6]. Adolescents who had a positive history of suicide attempts had significantly higher depression scores (93.7%) [7].

In the 1999 national survey of Egyptian children and adolescents, 59% of the sample reported experiencing feelings of fear or anxiety [8]. Forty per cent of children with anxiety disorders had a comorbid depressive disorder [9].

In the National Comorbidity Survey, most cases reported recurrent depressive episodes and significant role impairment, including attempted suicide among 21.9% of those with MDD [10]. Masked depression could be diagnosed in youths manifesting hyperactivity, aggressive behavior, or delinquency if they displayed depressed affect and showed depressive or pessimistic themes on projective tests [11].

Dysphoria and/or irritability may take the place of contentment and euthymia as the child's predominating mood state. Increasing levels of unhappiness, tearfulness, anger reactions, or frank rages set off by minimal or minor provocations may be noticed [12].

High levels of environmental stress as well as a few key stressful events were associated with suicide attempts; a recent romantic breakup or being assaulted added to suicide attempt risk, beyond the effects of psychopathology [13].

Rarely do adolescents seek, on their own, contact with mental health professionals for evaluation of developing mood symptoms, although they may more frequently make contact with available professionals or services located on-site in school settings. Adolescents disclose their depressive feelings more often through self-reports than to their parents [14].

### Aim of the study

Through this study, the authors aimed:

1- To estimate the prevalence of depressive disorders in Egyptian adolescent female students.

2- To estimate the characteristic symptom profile of Egyptian adolescent female students (if there is a characteristic profile).

### Hypothesis

The hypothesis of this study was that depressive disorders are highly prevalent among Egyptian female adolescents, and that there is a specific symptomatology characterizing the depressive disorders in this age group. However available literature provides no guidance in this matter.

## Methods

### Design of the Study

This study is a descriptive, cross-sectional, school based study.

### Site of the Study

This study was conducted in Cairo, Egypt. A sample of female adolescent school students in Eastern Cairo was drawn. Six schools were selected from two educational districts, one district represent higher socio-economic status (3 schools) and the other less affluent status (3 schools). From each school, 3 classes were selected and all students in each class were included.

### Selection

Basically a method of sampling was followed allowing each relevant factor to contribute in the constitution of the sample a share that was proportionate to its weight in the parent population. Determination of the size of this sample was done after the consultation of a statistician, sampling was performed randomly at five levels:

1- The city (Cairo) has 5 major geographical areas from which one was selected (Eastern Cairo).

2- Educational system in Eastern Cairo was divided into two major categories (Private and Public) based on socioeconomic profile.

3- From each category three schools were chosen.

4- Schools were chosen from two educational districts, one represents higher socioeconomic status (private schools) and the other less affluent status (public schools), those districts were (Heliopolis and El-Zaytoun).

5- From each school, 3 classes (one class represents each secondary grade) were selected and all students in each class were included. Selection of the classes was determined by the school authorities.

### Ethical considerations

During the time of data collection there was no ethical committee (recently established in Ain Shams University), however; the authors received the acceptance of authority figures in Ain Shams University and the Ministry of Education before starting the study procedures. In addition, an informed consent was obtained from each participant; they were informed about the questionnaires being used in the study and accepted their sharing in the study.

### Procedures

The data were collected by direct interviewing of the subjects in suitable settings inside their schools during a period from the beginning of November 2006 to the end of March 2007. At the time of the analysis, a total number of 602 adolescent female students participated in the study, while the number of non-participating female students was about 74 students. The apparent reason for non-participation was their absence from school at the period of the study or being missed during lessons or the period of the break.

The subjects of the study completed the following tools:

1- The General Health Questionnaire (GHQ), it is a screening instrument for psychiatric illness in order to identify potential cases which could then be verified and the nature of which could be determined by using a second stage instrument as it shouldn't be used as a sole criterion for diagnosis, it is mainly used to detect caseness [15]. The version used in this study is the Arabic version of a short 28-items scale with the sample scorer method which is (0-0-1-1) [16]. The cut-off point of GHQ was 7 according to similar previous national studies to minimize the associated fallacies with the original low threshold score [17]

2- The Children Depression Inventory (CDI), which is designed to be used as a screening instrument for depression in a normal adolescent sample or as a measure of symptom severity. It is useful for providing the clinician with structured, age and gender norm-referenced information about the child symptomatology [18]. The scale is suitable for children and adolescents from seven to eighteen years old. It consists of 27 groups of statements; every group consists of three statements representing the subject's feeling at the last two weeks. The score is from 0-2 according to the symptom severity and the total score ranges between 0-54. The cut-off point used for this study was 24 as similar previous national studies [19]. It has been standardized and translated to Arabic language [20]. Adolescents who scored more than 24 on (CDI) were further evaluated by the following questionnaires:

3- Structured Clinical Interview for DSM-IV Axis I Disorder (clinician version) (SCID-I), a semistructured diagnostic interview based on an efficient but thorough clinical evaluation [21]. The study used the Arabic version of the Structured Clinical Interview for DSM-IV axis I Disorders (SCID-I) [22].

4- The Hamilton Rating Scale for depression (Ham-D) designed to measure the severity of depressive symptoms in patients with primary depressive symptoms, it is the most commonly used observer rated depressive symptoms rating scale. Its internal consistency (Cronbach's alpha) was 0.76 [23], and 0.92 [24]. It is a checklist of items that are ranked on a scale of 0-4 or 0-2. Scoring: very severe >23, severe 19-22, moderate 14-18, mild 8-13 and normal < 7 [25].

### Statistical Analysis

All data were recorded and transferred on Statistical Package for Social Sciences (SPSS) Version 15. The results were tabulated, grouped and statistically analyzed using the following tests:

• Descriptive statistics were reported as means and frequencies.

• Pearson Chi square test (X^2^): to detect whether there is a significant association between different categorical variables.

• Student t-test: used to test for statistical significance of variance between two sample means.

• P value: used to indicate the level of significance: significant is P < 0.01.

## Results

The mean age for the studied sample was 15.7 + 0.9 years and 15.4 + 0.99 years for higher and lower social class schools respectively. A percentage of 15.3% of the studied sample were estimated to meet criteria for depression according to the CDI cut-off point. While, by the use of SCID-I about 13.3% of the studied population was found to have depressive disorders, distributed as 5% sub-threshold depressive symptoms, 5% MDD and 3.3% dysthymic disorder.

According to Ham-D, 10% of depressed female adolescents included in the study were classified as having moderate depressive state, while 30% had mild depressive state and 60% of them had subthreshold depressive state (Table 1).

**Table 1:** distribution of severity of depression among depressed students, as measured by Ham-D.

Ham-D Grades	Higher SES Depressed Students N = 39		Lower SES Depressed Students N = 41		X^2^	df	P	Sig
Moderate Depressive State	4	10%	4	11%	2.038	2	0.361	NS
Mild Depressive State	14	36%	9	21%				
Sub-threshold Depressive State	21	54%	28	68%				

In this study the fatigue or lack of energy (detected by Ham-D) was by far the most common symptom among depressed female adolescents (81.3%) followed by pessimism regarding the future, feeling sad, low self esteem, psychomotor retardation, lack of concentration, guilt, suicidality, insomnia, anhedonia, hypersomnia, weight gain, and lastly weight loss and psychomotor agitation (Table 2).

**Table 2:** Distribution of depressive symptoms among depressed students, as measured by SCID-I.

	Number of Adolescent Females	Percentage
Sad mood	59	73.8%
Anhedonia	35	43.8%
Weight loss, decreased appetite	24	30%
Weight gain, increased appetite	27	33.8%
Insomnia	36	45%
Hypersomnia	27	33.8%
Psychomotor agitation	16	20%
Psychomotor retardation	50	62.5%
Fatigue	65	81.3%
Decreased self esteem	59	73.8%
Pessimism	60	75%
Guilt	42	52.5%
Lack of concentration	47	58.8%
Suicidality	39	48.8%

This study revealed that 75.5% of adolescent females rated as having moderate depressive state had suicidal symptoms (detected by Ham-D), 52% of adolescent females rated as mild depressive state experienced the same symptoms, and 43% of students with subthreshold depressive state also had suicidal symptoms.

Suicidal ideations (answer 3 for the question about suicide in Ham-D) were the most common of the suicidal symptoms in adolescent females, 20% of the sample of depressed female adolescents, while the percentage of serious attempts was 2.5% of the sample.

Regarding comorbidity between depressive disorders and other psychiatric disorders (assessed by SCID-I) generalized anxiety disorder was the most prevalent comorbid diagnosis (32.5% of depressed students), followed by social phobia (20%) then substance abuse (8.8%) then obsessive compulsive disorder (0.1%) (Table 3). In addition screening by GHQ revealed minor psychiatric morbidity in 46.4% of adolescent females.

**Table 3:** Distribution of comorbid psychiatric diagnoses among depressed adolescent females according to SCID-I.

	Frequency	Percentage
Generalized Anxiety Disorder	26	32.5%
Social Anxiety Disorder	16	20%
Obsessive Compulsive Disorder	1	0.1%
Substance Abuse	7	8.8%
No comorbidity	30	37.5%
Total	80	100%

## Discussion

### I-Prevalence of depression

A percentage of 15.3% of the studied sample were estimated to meet criteria for depression according to the CDI cut-off point. The study answered the first part of the main hypothesis of the study. It revealed the point prevalence of depressive disorders among this sample of adolescent females according to SCID-I to be about 13.3%. This prevalence is relatively high when compared to similar studies. Kessler and Walters examined adolescents and young adults and found the 30-day prevalence was 5.8% (major depression) and 2.1% (minor depression) according to DSM-IV [10].

Higher prevalence of depressive disorders among adolescent females in an Egyptian community may be the result of a background of cultural, social and emotional instability characterizing this specific age group in addition to the discrimination against females prevailing societies in most of the third world countries sometimes declared and most of the time denied, in an attempt to wear civilized manners and behaviors.

### II-Symptoms

The second part of the main hypothesis was a trial to illustrate a specific symptomatology characterizing depression among adolescent females. The symptomatology characterizing depression in adolescent females was predominated by fatigue and lack of energy (more than 80%), sometimes with psychomotor retardation (about 2/3 of the sample). Also pessimism, sadness and low self esteem were expressed (about 3/4 of the sample). Insomnia was reported (45%) commoner than hypersomnia (33.8%). Weight gain and weight loss were reported, both were experienced almost equivocally (about 1/3 of the sample for each). Suicidality was found to be relatively high (about 1/2 of the sample).

### Somatic symptoms

In this study the fatigue and lack of energy were by far the most common symptoms among depressed female adolescents (81.3%), in addition to psychomotor retardation (62.5%), and psychomotor agitation (20%). This was in accordance with results of older Egyptian studies. The clinical profile of psychiatric disorders (DSM-III and III-R respectively) in the Egyptian community was previously studied [26,27] and they found that somatic symptoms were the most common symptom, among the depressed Egyptian population. The results were nearly similar to that of Torros et al (2004) who found that the most common depressive symptoms (measured by CBDI) were fatigue and somatic symptoms in a sample of Turkish adolescents.

However; these results were different from those of other studies performed in western countries, the most common symptoms among depressed adolescents were feelings of sadness, joylessness [28], depressed mood and sleep disturbances [14].

The discrepancy between eastern and western communities as regards the way the adolescent females experience and express their depression may be an interesting area for future researches.

In Egyptian culture, people tend to mask their affect with somatic complaints, which occupy the foreground and the affective component of their illness recedes to the background. This may be due to greater social acceptance of physical complaints than of psychological complaints which are either not taken seriously or are believed to be cured by rest or praying. Physical illness and somatic manifestation of psychological distress are more acceptable and likely to provoke a caring response than the vague complaints of psychological distress which can be disregarded or considered as a weakness or a degree of insanity [29].

A recent study performed by Stein et al (2010) examined ethnic/racial differences at the start of treatment among participants in the Treatment for Adolescents with Depression Study (TADS). African American and Latino youth were compared to Caucasian youth on symptom presentation and cognitive variables associated with depression. Contrary to hypothesis, there were no significant differences in symptom presentation as measured by the interview-based items of the Children's Depression Rating Scale-Revised (CDRS-R) [30].

### Emotional symptoms

Adolescent females in this study showed a range of emotional and cognitive symptoms in the context of depressive symptoms. Pessimism (75%), sadness (73.8%), and low self esteem (73.8%) were the commonest (Table 2).

The self perceptions of depressed adolescents usually are marked by feelings of inadequacy, inferiority, failure, and worthlessness. Evaluation of this criterion is challenging because many teens do not directly acknowledge such negative self perceptions. Many adolescents directly report a depressed mood much of the time, however; depression in adolescents commonly expresses itself as an irritable mood, because many adolescents lack the emotional and cognitive sophistication to correctly identify and organize their emotional experiences [31]

In accordance to the current study, Montague et al (2008) indicated a strong relationship between depressive symptoms and self-concept. Compared with the other groups, adolescents in special education at risk for emotional and behavioral disorders showed a significant decrease in self-concept after age 15. Additionally, high internalizing behavior was associated with more depressive symptoms and lower self-concept [32].

Although strongly prohibited by the Egyptian community, romantic relationships and failed love affairs may play a major role in the etiology of depressive symptoms among adolescent females. These adolescents have to face their frustrations and fix their own mistakes either alone or seeking the help of the inexperienced friends and peers.

### Vegetative symptoms

Adolescent females in this study had vegetative symptoms ranging between typical and atypical symptoms of depression: (45%) suffered from insomnia, while (33.8%) experienced hypersomnia. Review of literature revealed different trends: a tendency to describe atypical depressive symptoms in the adolescent age groups. This may be attributed to the identity confusion and rebelling attitude towards traditions and norms implied by the family and the society, leading to obvious changes in the sleep pattern and rhythm. Sleep disturbance is common in depressed adolescents,(interviewed by the Schedule for Affective Disorders and Schizophrenia for School-Age Children-and completed the mood and feelings self-report depression questionnaire) many of whom describe their sleep as non restorative and report difficulty getting out of bed in the morning. Sleep disturbance manifests as insomnia, hypersomnia or significant shifts of sleep pattern over the diurnal cycle [33]. These findings were not in accordance with the current study. Detailed analysis of the sleep pattern should be evaluated in further research.

As regards weight changes, weight gain was a symptom in (33.8%) of the depressed females, and weight loss was experienced by (30%) of them (Table 2). The adolescents are showing an overconcern with their physical appearance which is usually the result of their conformity to peer group influence. Other studies showed different results about appetite and weight changes: anorexia is more prevalent in adolescent girls. While some adolescents with depressive disorders crave and eat more specific foods (i.e. junk food and carbohydrates) and accordingly gain more weight than expected during their adolescent growth spurt [34]. Further evaluation of appetite and weight changes among depressed adolescent females is needed.

### Suicidality

In this study suicidal symptoms (including death wishes, suicidal ideation and suicidal attempts) were declared by (48.8%) of depressed adolescent females. The frequency of suicidal symptoms was 75.5% among subjects with moderate depressive state (8 subjects). While of the adolescent females suffering of mild depressive state (23 subjects), 52% experienced suicidal symptoms. Interestingly, of the subjects who experienced subthreshold depressive state (49 subjects) 43% also experienced suicidal symptoms. These findings are higher than the findings of other studies but in accordance with them: One of these studies showed that 35% of depressed adolescents had suicidal symptoms [35], Another study showed the rate of suicidal symptoms to be 30% among depressed students [31] while a third study revealed attempted suicide among 21.9% of the adolescents with major depression [10]. Another Egyptian study revealed that suicidal ideation and attempts were common among depressed Egyptian adolescents, 30% of the sample reported that they had strong death wishes (measured by CDI) or had a plan to harm themselves [19].

The rate of suicidality in the current study was much higher than the rate detected by Torros et al (2004) who found that suicidal symptoms (measured by CBDI) were positive in 6.9% of depressed Turkish adolescent girls [36], this variation in results may be explained by cultural and religious differences resulting in underreporting of suicidal symptoms due to fear of shame or guilt. The current study revealed that depressed adolescent females may have suicidal symptoms, regardless the severity or number of depressive symptoms. This finding shades light on the fact that the subthreshold depressive symptoms in adolescence -not merely clinical depression-should be taken seriously. Subjects with subthreshold depression should not be classified as "non-cases" neither to be treated as though they have a similar prognosis to those who are asymptomatic.

A study performed by Fordwood et al (2007) examined suicide attempts among depressed primary care adolescent patients, youth classified as suicide attempters showed elevated levels of psychopathology, specifically depressive symptoms, externalizing behavioral disorders, anxiety substance use, mania and PTSD symptoms [13]. Further evaluation of suicidality in this specific age group need to be done in future research.

### III-Psychiatric comorbidity

In this study, generalized anxiety disorder was the most prevalent co-morbid diagnosis among depressed adolescent females, in addition to social anxiety disorder, and substance use (Table 3). This is consonant with other studies which showed that anxiety disorder was the most common comorbid disorder with depression [10,37-39].

In the study conducted by Chaplin and Gilham (2009), total anxiety and worry and oversensitivity symptoms were found to predict later depressive symptoms more strongly for girls than for boys. Physiological anxiety predicts later depressive symptoms for both boys and girls. These findings which are consonant with the results of the current study, highlight the importance of anxiety for the development of depression in adolescence, particularly worry and oversensitivity among girls [40].

### Strengths and limitations

As one of the few studies that have investigated the prevalence and the symptomology of adolescent depression, the present study has employed a comprehensive battery of psychiatric tools for screening, diagnosis and assessment of severity of depression, the use of a semi-structured clinical interview for diagnosis, not relying on the self-reports. The interviews used were directly addressed to students not in presence of their families which is more relieving to female adolescents, they prefer to talk about their feelings in their peer environment rather than in front of parents.

Despite all of these strengths, there are some limitations of this study that require careful consideration in the interpretation of the findings. First, the size of the sample was relatively small. Second, the psychiatric diagnoses were mainly based on clinical interviews of study subjects without interviewing their parents. Previous studies have shown low agreement among child, parent, and teacher informants in reporting children's emotional and behavioral problems and the need to incorporate teachers' reports into the identification of depressive symptomology. Third, a more comprehensive study would add a tool for a detailed personality assessment, to exclude the effect of some personality traits on the subjects' behavior, e.g.suicidality. Fourth, the estimation of suicidality was based on questions within the Ham-D, this would better be assessed by a specific scale for suicidality. Finally, the setting for the study, as it is known that administrating self reports in non clinical population may result in inflated scores. The lack of complete information in psychiatric diagnoses for all study subjects has impeded the possibility for detailed longitudinal analyses of psychiatric symptoms.

## Conclusions

The somatic symptoms were by far the most common presenting symptom for female adolescents suffering from depressive disorders. Depressive phenomena including unexplained fatigue, decreased energy, psychomotor changes, lack of concentration, weight changes and suicidal ideations may be the presenting complaints instead of the classic sad mood.

Further studies are needed to check if early detection of depressive disorders in adolescents may affect the course of the depressive illness, and its complications i.e. Substance abuse, scholastic deterioration and suicidality. Further investigation of risk factors, longitudinal course of depressive symptoms, level of functioning, patterns of comorbidity, and the psychopathological background of adolescent population at risk would be completing the picture in this area of research.

## Competing interests

The authors declare that they have no competing interests.

## Authors' contributions

AHK, suggested the idea of this research and facilitated the study procedures, she made substantial contributions to the conception, the design and the methodology. She revised the results and the discussion, and was involved in the last corrections conducted in response to the reviewers' comments. MAR, has been involved in the conduction of clinical interviewing, collecting references, revising the manuscript critically for intellectual content, performed the final revision and approval of the manuscript, and was involved in the last corrections conducted in response to the reviewers' comments. MFA, has been involved in the design and the methodology of the study, the conduction of clinical interviewing, the interpretation of data, and the critical revision of the manuscript. TAA, managed to provide the rest of authors with most of the references, he was involved in the study design and methodology, revised the results and the discussion, provided the training for using SCID-I prior to research. AHE, have been involved in drafting the manuscript, substantial revision and clarification of the methodology, results and conclusion, provided the training for using Ham-D prior to research. WMS, has made substantial contribution to design and methodology, acquisition and interpretation of data. She has been involved in drafting the manuscript. All the above mentioned authors read and approved the final manuscript.
